# Supplementary material for: Filling the gaps: leveraging large language models for temporal harmonization of clinical text across multiple medical visits for clinical prediction
Source: medRxiv. 2024 May 7:2024.05.06.24306959. Preprint. [Version 1] doi: 10.1101/2024.05.06.24306959 (PMC11100936; doi:10.1101/2024.05.06.24306959)
Supplement: Supplement 1 [file NIHPP2024.05.06.24306959v1-supplement-1.pdf]

medRxiv preprint doi: https://doi.org/10.1101/2024.05.06.24306959; this version posted May 7, 2024. The copyright holder for this preprint (which was not certified by peer review) is the author/funder, who has granted medRxiv a license to display the preprint in perpetuity.
It is made available under a CC-BY 4.0 International license .

Supplementary material:

Table S1: Performance metrics of supervised learning methods by temporal harmonization method including PPV and Recall

	BiLSTM				Transformer			
	**AUC**	**PPV**	**Recall**	**F1 score**	**AUC**	**PPV**	**Recall**	**F1 score**

medRxiv preprint doi: https://doi.org/10.1101/2024.05.06.24306959; this version posted May 7, 2024. The copyright holder for this preprint (which was not certified by peer review) is the author/funder, who has granted medRxiv a license to display the preprint in perpetuity.
It is made available under a CC-BY 4.0 International license .

**Zero pad**	0.739	0.671	**0.764**	0.715	0.797	**0.824**	0.318	0.460
**LOCF**	0.748	0.689	0.671	0.679	0.791	0.756	0.568	0.649
**Multimodal Imputation**	0.683	0.617	0.669	0.642	0.725	0.696	0.331	0.448
**Biomistral (zero shot)**	0.739	0.645	0.742	0.690	0.770	0.776	0.452	0.571
**Biomistral (one shot)**	0.733	0.671	0.678	0.674	0.777	0.651	0.793	0.715
**Asclepius (zero shot)**	0.741	0.663	0.720	0.690	0.769	0.737	0.566	0.640
**Asclepius (one shot)**	0.742	0.695	0.646	0.669	0.774	0.682	**0.776**	0.726
**GPT-4 (zero shot)**	**0.785**	**0.713**	0.736	**0.723**	**0.817**	0.790	0.638	0.706
**GPT-4 (one shot)**	0.777	0.709	0.703	0.706	**0.817**	0.767	0.703	**0.734**

Table S2: Quantifying improvements in performance for patients with more or less missing data including PPV and Recall

		**BiLSTM**				**Transformer**			
		AUC	PPV	Recall	F1 score	AUC	PPV	Recall	F1 score
**Missing < 50%**	**Zero pad**	0.749	0.727	0.697	0.712	0.801	0.826	0.326	0.467
	**GPT-4 (zero shot)**	0.809	0.747	0.747	0.747	0.808	0.728	0.759	0.744
	**GPT-4 (one shot)**	0.761	0.715	0.705	0.711	0.801	0.771	0.683	0.724
**Missing > 50%**	**Zero pad**	0.728	0.670	0.676	0.673	0.781	0.753	0.509	0.608
	**GPT-4 (zero shot)**	0.846	0.757	0.722	0.739	0.818	0.731	0.704	0.717
	**GPT-4 (one shot)**	0.813	0.681	0.750	0.717	0.836	0.800	0.629	0.705
